# On the Purity of Foreign Iodide of Potassium

**Published:** 1864-12

**Authors:** F. C. Clayton


On the Purity of Foreign Iodide of Potassium.—By F. C. Clayton.—The high price and large consumption of this article has made it one which the manufacturer has special temptations to adulterate. Of late years very large quantities of foreign make, have found their way into our markets, giving rise to keen competition, which in the case of drugs, is often far from improving their quality. From these considerations we might still expect to find much that is impure, but the results detailed below lead us to a different conclusion. The impurities of iodide of potassium are bromide and chloride of potassium, and sulphate, iodate, and carbonate of potash. Moisture in excess is also to be considered an impurity, for, besides giving the sample a greater liability to deliquesce, it shows an article of imperfect manufacture. The first mentioned adulterant, though it has at times been frequently used, has in none of the fifteen samples experimented upon been found, and the second only in quantities of 3-7 per cent. down to minute traces. Sulphate was never found in ponderable quantities, and iodate in only 3, all of which, however, were of foreign manufacture. (Several English samples were analysed for the sake of comparison). In these three cases it never amounted to 1 per cent. Carbonate, though more generally present, never amounted to 1 per cent., generally much under this. From these results, it will be seen that the iodide of potassium now in the market is practically pure, the percentage in all the samples being over 93°.—*Journal of Pharmacy.*

